# Application and prospect of organoid technology in breast cancer

**DOI:** 10.3389/fimmu.2024.1413858

**Published:** 2024-08-26

**Authors:** Shanlin Huang, Zifan Mei, Andi Wan, Min Zhao, Xiaowei Qi

**Affiliations:** ^1^ Department of Breast and Thyroid Surgery, Southwest Hospital, Army Medical University, Chongqing, China; ^2^ Key Laboratory of Chongqing Health Commission for Minimally Invasive and Precise Diagnosis and Treatment of Breast cancer, Southwest Hospital, Army Medical University, Chongqing, China

**Keywords:** organoids, breast cancer, drug screening, personalized treatment, tumor research models

## Abstract

Breast cancer is the most common malignant tumor in women. Due to the high heterogeneity of breast cancer cells, traditional *in vitro* research models still have major limitations. Therefore, it is urgent to establish an experimental model that can accurately simulate the characteristics of human breast cancer. Breast cancer organoid technology emerged as the times required, that is, to construct tissue analogs with organ characteristics by using a patient’s tumor tissue through 3D culture *in vitro*. Since the breast cancer organoid can fully preserve the histology and genetic characteristics of the original tumor, it provides a reliable model for preclinical drug screening, establishment of breast cancer organoid biobanks, research into the mechanisms of tumor development, and determination of cancer targets. It has promoted personalized treatment for clinical breast cancer patients. This article mainly focuses on recent research progress and applications of organoid technology in breast cancer, discussing the current limitations and prospects of breast cancer organoid technology.

## 1 Introduction

Breast cancer has the highest incidence rate among malignant tumors in women worldwide and is also the primary cause of cancer-related death in women, accounting for 23% of female cancer deaths (1, 2). Currently, clinically, breast cancer is classified and treated differently based on the expression differences in the estrogen receptor, progesterone receptor, and human epidermal growth factor receptor in patients (3–6). Based on their molecular and genetic characteristics, breast cancers are generally classified into five subtypes: luminal A, luminal B, HER2-positive, triple-negative A, and triple-negative B (7). However, due to the high heterogeneity of breast cancer at morphological and molecular levels, invasive nature, and drug resistance, traditional treatment regimens do not apply to all patients. 40% of breast cancer patients experience tumor recurrence, and among them, 60%-70% develop distant metastases (8–10). Next-generation sequencing provides genomic information for patients, creating opportunities for personalized cancer treatment and guiding treatment decisions (11, 12). However, targeted drugs that meet the detected tumor genetic data are still very limited, most lack preclinical data and reliable drug descriptions, thus the number of patients who can benefit from this method is extremely limited (13, 14). Traditional research models for breast cancer, such as *in vitro* cultivation of tumor cell lines and organ tissue section culture, lack the *in vivo* three-dimensional tumor microenvironment (15). In addition, animal models have unavoidable human-mouse species segregation, which has become an obstacle to *in vitro* study of tumor cell drug resistance, high-throughput gene sequencing, and the development of precise therapeutic regimens (16). Therefore, the development of clinical precision medicine urgently needs *in vitro* models that can well-simulate the unique molecular subtypes of patients.

Organoids preserve the molecular, structural, and functional characteristics of their tissue of origin, thus demonstrating great potential in studying the biology of human tissues in health and disease (17). Experts from 16 countries have collectively defined organoids as entities derived from (pluripotent) stem cells, progenitor cells, and/or differentiated cells that self-organize through cell-cell and cell-matrix interactions, summarizing various aspects of *in vitro* natural tissue structure and function (18). Organoids combine cells with varied differentiation potentials (e.g., embryonic stem cells, pluripotent stem cells, and tumor cells) with *in vitro* three-dimensional cultivation techniques. This involves the introduction of extracellular matrix analogs and cytokines to maintain stem cell proliferation and differentiation. This model can not only effectively mimic the *in vivo* environment, forming cell tissue structures similar to the source organs in the body, but it can also achieve functional reproductions that traditional *in vitro* cultivation systems cannot attain (19). In addition, most of the cells cultured in organoids are derived from the patient’s own stem cells and tumor cells, which avoid ethical controversy and species isolation to a certain extent and can better conduct pathogenesis research and drug screening for individual tumor heterogeneity, genetic testing, and personalized treatment (20, 21).

Bibliometrics is a method of document analysis that evaluates the internal connections and distribution patterns among research documents from quantitative and qualitative perspectives. Its purpose is to gain insight into the current state of study, research hotspots, and future trends in a particular field (22). When combined with visual analysis, bibliometrics becomes an effective tool for integrating information and enhancing understanding of the research process (23, 24). Therefore, we conducted a bibliometric analysis of relevant literature on breast cancer organoids obtained from the Web of Science Core Collection (WOSCC) database. This analysis aims to provide directions for future research.

## 2 Bibliometric analysis

We conducted a comprehensive search and analysis of organoid- and breast cancer-related literature using bibliometric statistical methods by searching the Web of Science Core Collection (WOSCC). The retrieval strategy used the keywords “organoid* (Topic) AND breast cancer* or Breast Neoplasm* or Mammary Cancer* or Breast Malignant Tumor* or Mammary Carcinoma* or Mammary Neoplasm* or Breast Carcinoma (Topic)”. All published articles were analyzed without year, language, or source (article, book, etc.) filtering to include all content published on the topic. All data were retrieved at the same time (July 2024) to avoid biases caused by database updates. A total of 874 articles were obtained. The publication count, citation count, and research trends of countries, authors, and institutions were visually analyzed using CiteSpace (version 6.2.R4), VOSviewer (version 1.6.18), and the bibliometric package in R language.

### 2.1 Overall status of breast cancer organoid research

#### 2.1.1 Analysis of annual publication volume and trends

**Figure 1:**
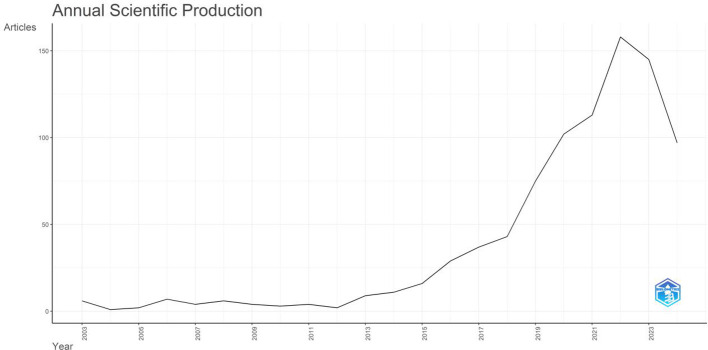
Annual publication volume and publication trend in the field of breast cancer organoid research in the WOS core collection.

Academic research generally reflects the current research hotspots and clinical needs. In this study, a total of 874 articles met the search criteria. **Figure 1** depicts the total number of papers published each year, with an increasing trend from six articles in 2003 to 156 articles in 2021. The initial phase of breast cancer and organoid research was between 2003 and 2012 (average annual publication volume: 3.8). The number of published research articles increased significantly after 2012. By December 31, 2022, the annual growth rate was 54.6%. The results suggested that the organoid- and breast cancer-related fields are receiving increasing research attention.

#### 2.1.2 Analysis of core researchers and research institutions

CiteSpace software enables visualization of the important researchers in breast cancer organoid research. The results demonstrated that 5,210 authors have been involved in organoid and breast cancer research. The data were analyzed using R software. The g-index is a derivative of the h-index that was introduced mainly to compensate for the fact that the h-index does not reflect highly cited papers well. The g-index is defined as follows: for papers sorted by citation count, the maximum rank g refers to the top g papers cited at least g^2^ times, i.e., the (g + 1) order. The article corresponding to Clevers H had the highest g-index of 17, followed by the articles corresponding to Ewald AJ (g-index: 16), Skala MC (g-index: 9), Jiang YZ (g-index: 9), and Kim J (g-index: 9) (**Table 1**).

**Table 1:** Core researchers in the field of breast cancer organoid research in the WOS core collection.

Element	g_index	TC	NP	PY_start
CLEVERS H	17	4254	17	2016
EWALD AJ	16	979	16	2013
SKALA MC	9	532	9	2014
JIANG YZ	9	211	9	2020
KIM J	9	114	9	2017
LEE S	9	98	12	2015
ROSENBLUTH JM	9	230	9	2018
CHEN Y	8	734	8	2017
WELM BE	8	230	8	2013

**Table 2** demonstrates that 282 journals published articles on organoids and breast cancer from 2003 to 2024. The data were analyzed using R software. The US-based *Cancer Research* had the highest g-index of 34 and published the most articles (n = 68). This was followed by the UK-based *Nature Communications* (g-index: 26, 26 publications) and the Switzerland-based *Cancers* (g-index: 20, 234 publications).

**Table 2:** Top 10 journals and the most frequently cited journals in breast cancer organoid research.

Element	g_index	TC	NP	PY_start
CANCER RESEARCH	34	1186	68	2006
NATURE COMMUNICATIONS	26	1298	26	2016
CANCERS	20	448	34	2019
ONCOGENE	18	388	18	2005
SCIENTIFIC REPORTS	17	419	17	2016
PLOS ONE	15	607	15	2009
PROCEEDINGS OF THE NATIONAL ACADEMY OF SCIENCES OF THE UNITED STATES OF AMERICA	14	656	14	2009
CANCER SCIENCE	14	477	14	2008
CELL REPORTS	13	481	13	2015

#### 2.1.3 Countries and institutions

Organoids and breast cancer are being actively researched worldwide. Here, we report the status of published literature, where 44 regions/countries have published research on organoids and breast cancer. These regions/countries have a diverse array of collaborative teams.

**Figure 2:**
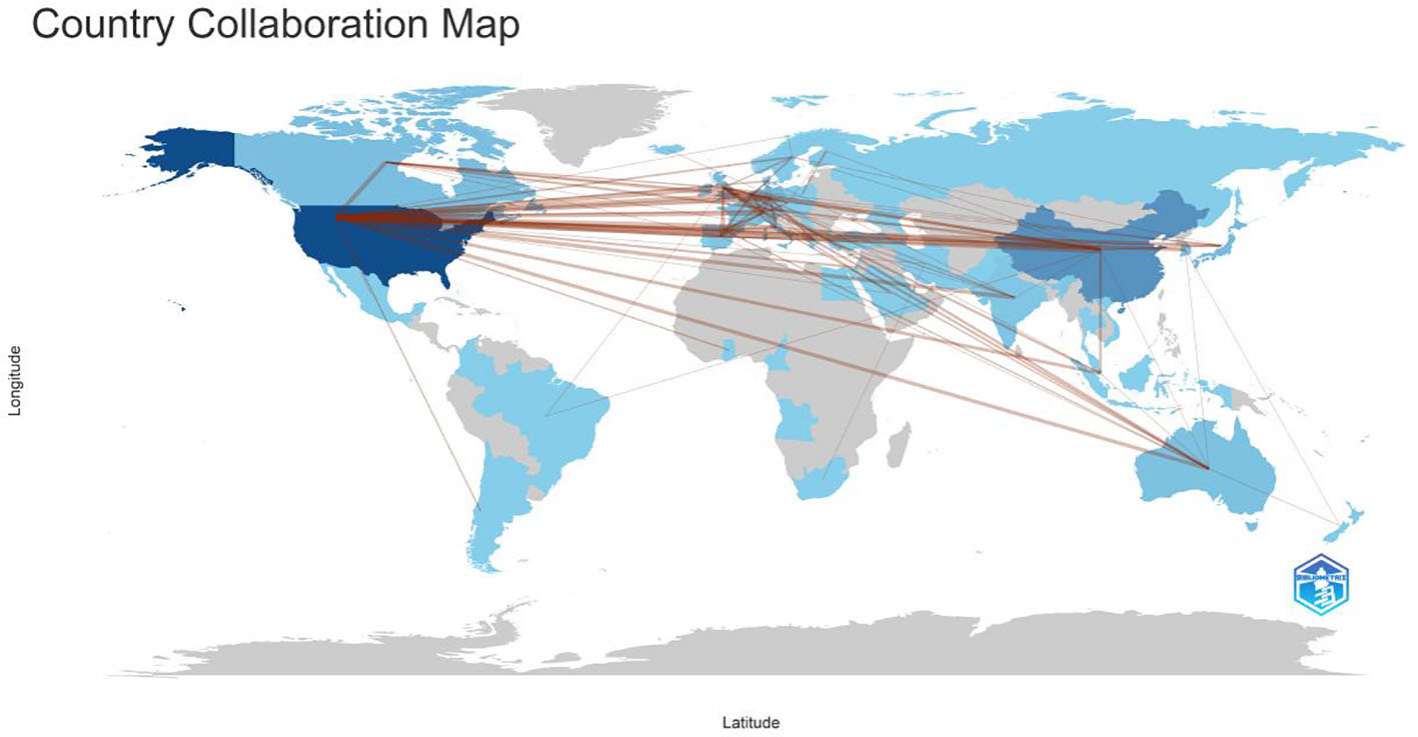
Countries’ collaboration world map in the WOS core collection about breast cancer organoid research. The red lines represents cooperation between countries.

Among the top 10 regions/countries in organoid and breast cancer research, the USA had 12,124 citations, followed by New Zealand (4,651 citations), Japan (1,608 citations), Canada (1,413 citations), and Australia (928 citations) (**Table 3**). New Zealand had the highest average citations per article (155.00 citations per article). There were many active collaborations between countries and regions. The USA had the most extensive collaborations with other countries and regions, including Europe, China, and Japan (**Figure 2**). Europe also closely collaborated with China. Among the 1,411 institutions conducting organoid and breast cancer research (**Table 4**), Harvard University published the most articles (113), followed by the University of California (82), Johns Hopkins University (69), and the University of Texas System (65).

**Table 3:** Analysis of regions in the WOS core collection related to breast cancer organoid research.

Region	Freq	TC	Average Article Citations
USA	1675	12124	38.9
CHINA	663	2243	15.2
ITALY	267	669	19.7
GERMANY	263	480	17.1
NETHERLANDS	194	4651	155
CANADA	186	1413	42.8
AUSTRALIA	184	928	37.1
JAPAN	173	1608	43.5
UK	138	724	27.8

**Table 4:** Institutions in the WOS core collection about breast cancer organoid research.

Affiliation	Articles
HARVARD UNIVERSITY	113
UNIVERSITY OF CALIFORNIA SYSTEM	82
JOHNS HOPKINS UNIVERSITY	69
UNIVERSITY OF TEXAS SYSTEM	65
UNIVERSITY OF TORONTO	65
HARVARD MEDICAL SCHOOL	55
HELMHOLTZ ASSOCIATION	55
MEMORIAL SLOAN KETTERING CANCER CENTER	53
UNIVERSITY OF CALIFORNIA SAN FRANCISCO	49

### 2.2 Evolution of research hotspots and frontier development trends

In bibliometrics, topics discussed relatively more frequently and are more closely related within a certain period can be considered hotspots in a certain field. Keywords reflect and summarize the overall content of the article. We evaluated the organoid and breast cancer research hotspots using CiteSpace keyword analysis and interpretation.

**Figure 3:**
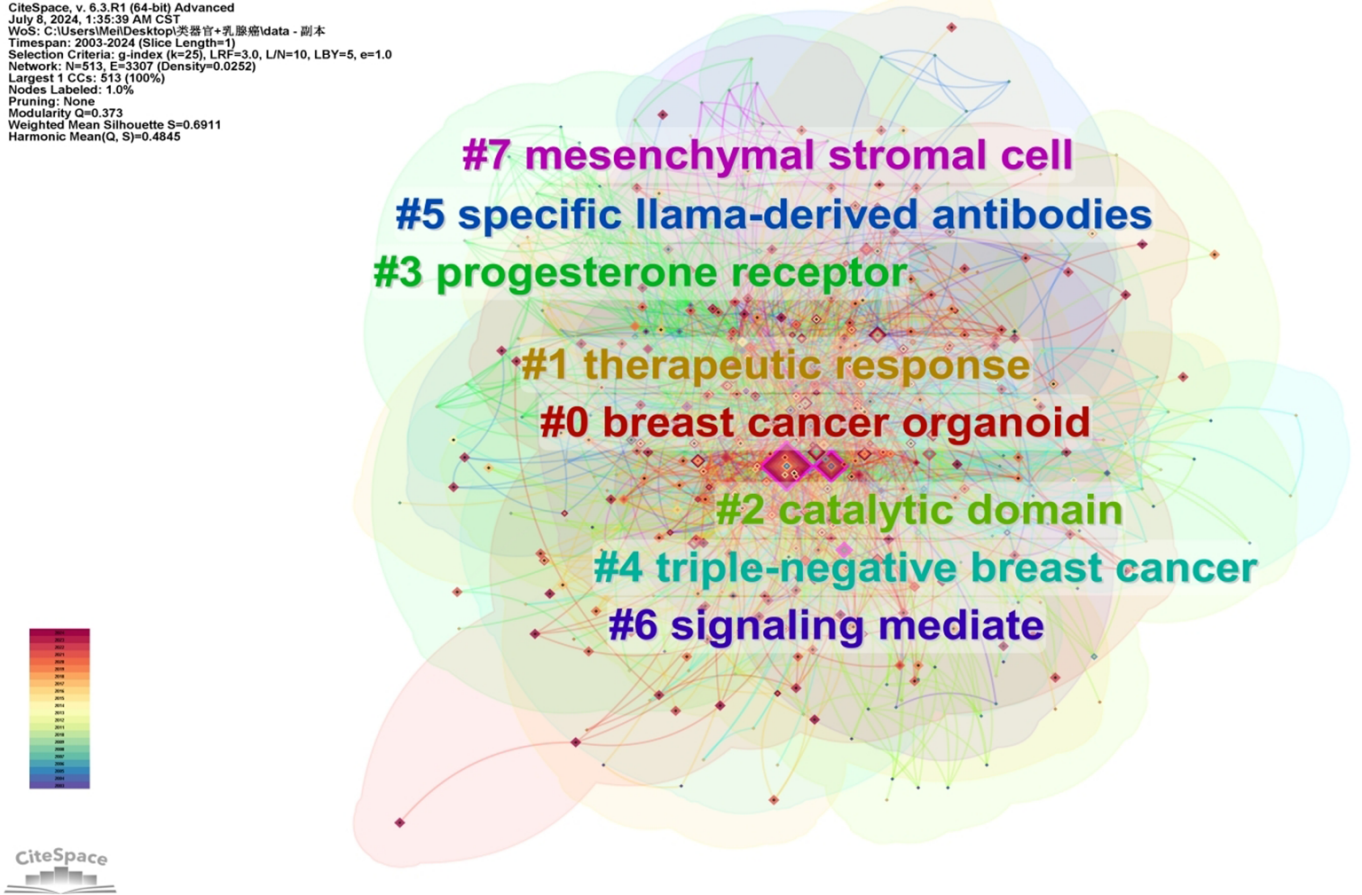
WOS core collection’s keywords’ clusters in breast cancer organoid research. Each cluster can to a certain extent reflect a focal area of interest within the field, and be ranked based on the level of attention in related research.

**Figure 4:**
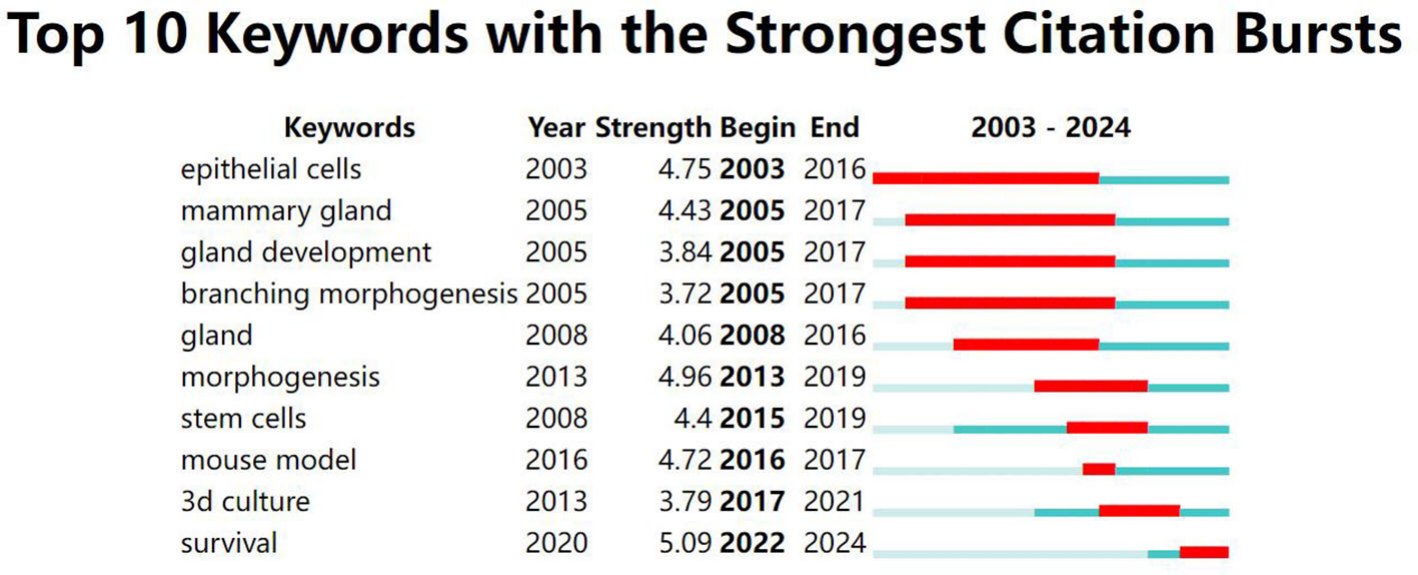
WOS core collection’s hotspots in breast cancer organoid research.

A keyword map was created using cluster analysis of the co-occurrence knowledge map of keywords. The top nine keyword groups from the analysis were “breast cancer organoid”, “therapeutic response”, “catalytic domain”, “progesterone receptor”, “triple-negative breast cancer”, “specific llama-derived antibodies”, “signaling mediate”, and “mesenchymal stromal cell” (**Figure 3**). Keywords with high citation frequencies are another important indicator reflecting research frontiers and hotspots. **Figure 4** demonstrates that researchers recently focused on “survival” (2022–2024), “3D culture” (2018–2022), “mouse model” (2019–2018), “stem cells” (2016–2020), and “morphogenesis” (2014–2020). This focus was ongoing, indicating that these research topics have received significant attention recently and may become the new frontiers.

## 3 Organoid technology

### 3.1 The concept of organoids

Organoids refer to the use of adult stem cells or pluripotent stem cells for three-dimensional culture *in vitro*, and under the action of growth factors and cytokines provided *in vitro*, differentiate to form tissue analogs with various cell types and spatial structures (24). Organoid technology can not only reproduce the structure and function of tissue, but also simulate the biological characteristics of tissue *in vivo*, so it is also called a “miniature organ”. Among them, tumor organoids use the patient’s tumor tissue to establish a living cell model, while maintaining a high degree of heterogeneity between tumor patients and within the tumor, and at the same time, it can individualize effective therapeutic drugs. In addition to guiding personalized medication, it can provide a more precise and direct treatment plan. This makes it the fourth preclinical model besides the two-dimensional cell culture, human tumor xenograft model, and organ tissue slice model, and has the advantages of the first three with a wide application value in the field of tumor research (25). Breast cancer organoids provide an ex vivo model that preserves certain cellular, structural, and microenvironmental characteristics, which determine the function of the breast *in vivo* and greatly enhance our understanding of gland biology. They are amenable to genetic manipulation, real-time imaging, and high-throughput screening, which aids in the study of gland morphogenesis, structural maintenance, tumor progression, and mechanisms of invasion (26).

### 3.2 Progress in culturing breast cancer organoids

The earlier CiteSpace results determined that the number of published research articles on breast cancer organoids increased significantly since 2012, and culture techniques for breast cancer organoids had been optimized during this period. Furthermore, bibliometrics indicated that “3D culture” was a high-citation keyword.

**Figure 5:**
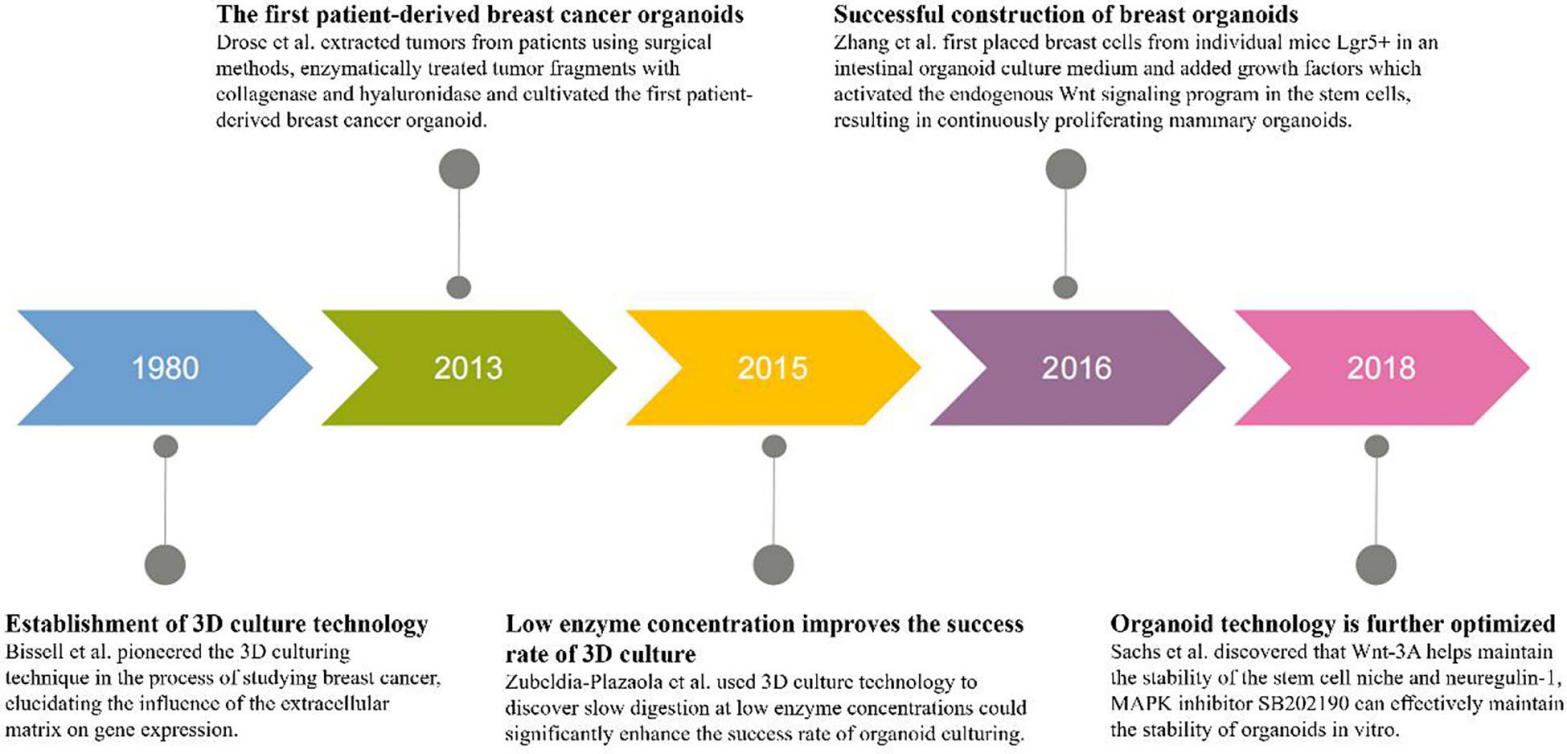
Timeline of breast cancer organoid culture progress. From left to right, the timeline depicts five different years and progress in culturing breast cancer organoids. Bissell and colleagues pioneered the 3D culturing technique in the 1980s. In 2013, Drose and associates cultivated the first patient-derived breast cancer organoid. In 2015, Zubeldia-Plazaola and colleagues discovered that slow digestion at low enzyme concentrations significantly enhanced the success rate of organoid culturing. In 2016, Zhang and co-workers successfully constructed a breast organoid. In 2018, Sachs and associates discovered the effect of neuregulin-1 and Wnt-3A on organoid growth, further developing organoid technology.

In the 1980s, Bissell and others pioneered the three-dimensional(3D) culturing technique in the process of studying breast cancer, elucidating the influence of the extracellular matrix on gene expression (**Figure 5**). In 2013, Drose and colleagues extracted tumors from patients using surgical methods, enzymatically treated tumor fragments with collagenase and hyaluronidase, and cultivated the first patient-derived breast cancer organoid (27) (**Figure 5**). Experimental results indicated that the digestion time and culturing conditions needed for tumor tissues from different sources varied. In 2015, Zubeldia-Plazaola and team used 3D cell culture technology to establish primary mammary epithelial cell lines more effectively, discovering that slow digestion at low enzyme concentrations could significantly enhance the success rate of organoid culturing (28) (**Figure 5**). In 2016, Zhang et al. first placed breast cells from individual mice Lgr5+ in an intestinal organoid culture medium and added growth factors like epidermal growth factor(EGF), exogenous ligands Wnt-3A of the Wnt signaling pathway, and R-spondin (**Figure 5**). This activated the endogenous Wnt signaling program in the stem cells, resulting in continuously proliferating mammary organoids. Under the induction of estrogen, the luminal cells spontaneously organized into ductal structures (29). In 2018, Sachs and others published a paper in “Cell”, where they collected samples from over 150 breast cancer patients, successfully producing over 100 organoids (**Figure 5**). Through Next-Generation Sequencing (NGS) data analysis, it was found that organoids could retain the copy number variants (CNV) and single nucleotide variants (SNV) characteristics of their paired tumor samples well. They discovered that neuregulin-1 is a ligand for human epidermal growth factor receptor tyrosine kinases-3 and -4, which can extend the growth of breast cancer organoids *in vitro*. Wnt-3A helps maintain the stability of the stem cell niche but has no evident effect on promoting the growth of organoids. Moreover, the mitogen-activated protein kinase(MAPK) inhibitor SB202190 can effectively maintain the stability of organoids *in vitro*, but when its concentration exceeds 1μmol/L, it reduces their formation efficiency (30).

To date, more research has continuously improved organoid culture techniques to achieve higher purity organoids. Overgrowth or contamination of normal epithelial cells is a major drawback of epithelial tumor cell organoid culture (31, 32), but pure cancer organoid cultures can be obtained by providing minimal or selective culture media to inhibit the growth of normal epithelial cells. For instance, mutations activating the Wnt/β-catenin signaling pathway are found in about 95% of colorectal cancer(CRC) cases. Pure CRC organoid cultures can be obtained by removing Wnt pathway stimulants, such as Wnt ligands and R-spondins, from the growth medium (33, 34). In the field of breast cancer organoid culture, Dekkers et al. provided an optimized, highly general protocol for the long-term culture of organoids from both normal human mammary tissue and breast cancer tissue. They also used Lipofectamine 2000, electroporation, or lentiviral methods for genetic manipulation, selective culture, and cloning of organoids, providing a good guide for the construction of current breast cancer organoid models (35). Data provided by Goldhammer N et al. indicated that the highest similarity between lesion tissue and ex vivo cultured organoids is in the early passages. Over the past few years, with the optimization of the digestion steps and continuous improvement of experimental techniques, the success rate of culturing breast cancer organoids has increased to about 80%, which will become an effective model for further breast cancer research (36). The Chinese consensus on the quality control standards for the tumor organoid diagnosis and treatment platform issued in 2022 pointed out that the tissue used for tumor organoid culture should be fresh and rich in a sufficient amount of active tumor cells, and the number of cells after digestion should be at least greater than 104, with a cell survival rate greater than 90%, before subsequent operations can be performed. Although different organoids have different growth cycles, organoid formation can generally be seen within 7 days, and they can be passaged and expanded within 3 weeks.

After successful cultivation of breast cancer organoids, it is necessary to identify from multiple dimensions, such as morphology, histopathology, and molecular genetics, whether tumor organoids can restore the morphological phenotype and tumor biomarkers of the original tumor (37), such as the most common tumor markers in breast cancer: estrogen receptor, progesterone receptor, and human epidermal growth factor receptor 2, to ensure the credibility of subsequent organoid drug sensitivity test results. The “Guidelines for the Preparation, Cryopreservation, Subculture, and Identification of Human Normal Mammary and Breast Cancer Organoids” provides specific methods for the identification of breast cancer organoids, starting with morphological observation under an optical microscope, followed by cell viability analysis to ensure that the cultured organoids are vigorous and of good quality. Then, methods such as hematoxylin-eosin staining(HE staining) and immunohistochemical staining are used to compare the histological characteristics of the organoids with the original tumor tissue, and finally, the molecular typing of breast cancer organoids is performed, thereby obtaining a more reliable *in vitro* model, providing new technical means for the basic research of breast cancer, precision treatment, and new drug development.

## 4 Comparison of other breast cancer research models with organoid technology

The bibliometric analysis (**Figure 4**) identified the recent high-citation keywords in breast cancer organoids: “3D culture”, “mouse model”, and “morphogenesis mouse”. Hence, scientists are expected to examine the significant advantages of organoid models over the 2D cell line model, mouse model, and organ tissue slice model.

### 4.1 Two-dimensional cell line model

Stem cells are a general term for primitive and undifferentiated cells with self-replication function and multi-lineage differentiation potential. At present, there are two culture modes of stem cells: two-dimensional(2D) culture and three-dimensional culture. Among them, two-dimensional cell culture refers to immersing stem cells in the culture medium, on the surface of ordinary glass or plastic petri dishes, along the two-dimensional flat extension growth. From basic monolayer culture and functional analysis to the increasingly developed cell-based cancer interventions, 2D cell culture plays a crucial role in cancer diagnosis, prognosis, and treatment. The traditional 2D cell line model has the advantages of simple culture, easy operation, and visual observation of the cell state (38). Gambardella et al. performed transcriptional analysis on 35,276 single cells from 32 breast cancer cell lines to generate a single-cell map. Through large-scale *in vitro* drug screening, they found that transcriptional heterogeneity allows cells with different drug sensitivities to survive in the same population (39). In 2018, Pirsko et al. found that the expression of breast cancer cell differentiation phenotype markers was affected by the components of the cell growth medium and two-dimensional cell culture should be used as a tool for phenotype research (40). However, tumor cells in 2D cell culture models undergo changes in morphology and signaling networks, gradually losing heterogeneity and failing to establish a tumor microenvironment to simulate the actual cellular environment of tumors, thus unable to accurately predict the efficacy of drugs *in vivo* (41). The 2D cell line is derived from a single cell, lacks the influence of tumor cell heterogeneity and stem cell niche, has poor stability and is prone to mutation, and cannot well simulate the tissue specificity of patient tumors, which is limited in further research on the occurrence and development of tumors.

### 4.2 Patient-derived xenograft model

The patient-derived xenograft (PDX) model involves transplanting fresh tumor tissue from patients into immunodeficient mice, cultivating human tumor tissues in these mice. Because the PDX model retains the characteristics of the original patient’s tumor, including gene expression profiles and drug responses, avoiding *in vitro* genetic drift and clonal selection, it can better mimic the tumor cell growth environment in humans (42, 43). They are widely used in preclinical cancer models and are considered to capture the histological, molecular, and intratumoral heterogeneity of human tumors more accurately than cell culture models (44). After decades of research, it has been found that drug screening results using the PDX model highly correlate with clinical outcomes, leading many research institutions and pharmaceutical companies to adopt the PDX model for cancer drug screening and assessment (45). PDX models can also serve as a platform for studying individual patients’ sensitivities to targeted drugs and guide our understanding of various aspects of tumor biology, including tumor clonal evolution and interactions with the microenvironment (46). PDX model provides valuable specimens in personalized treatment, exploring the mechanism of tumor development and potential therapeutic targets, and brings greater clinical benefits to patients (47). Additionally, optimized PDX implantation procedures and modern technologies have made the molecular landscape description of PDX more comprehensive and facilitated the use of PDX models (48).

Compared to organoid models, PDX models have a unique advantage in simulating the tumor microenvironment and maintaining cell heterogeneity. However, due to their long cultivation cycle, high costs, and ethical concerns surrounding animal use, they are not easily scalable for clinical research. Furthermore, the inherent species differences between mice and humans mean they can’t fully simulate human histology and genomic diversity, making them unsuitable for high-throughput drug screening (49, 50).

### 4.3 Organ tissue slice model

Organ tissue slice cultures offer a complete, natural microenvironment and extracellular matrix for studying tumor cell behavior. Many cell morphologies, such as immune cells, endothelial cells, and cancer cells, and structures like blood and lymph vessels, can easily be visualized in native tumor slices using fluorescently labeled antibodies (51). Compared to organoid technology, tumor tissue slice models from patients can maintain the tumor microenvironment in a short time, enhancing the sensitivity and accuracy of the preclinical drug *in vitro* screening and therapeutic decision-making (52). Tumor tissue slice models have proven to be a valuable model for clinical and basic research, especially when a study drug relies on multiple aspects of the breast tumor microenvironment, such as immune cells or extracellular matrix(ECM) composition, making slice culture more suitable for drug testing (53). However, organ tissue slice models cannot be preserved long-term. The retention of tissue morphology, viability, and cell proliferation varies from a few days to several months depending on the slice thickness, making them unsuitable for reflecting the potential of tumor stem cells and the complete tumor specificity (54).

### 4.4 Unique advantages of organoid models

In the last decade, the development of organoids has rapidly advanced, and three-dimensional cell model organoid technology has gradually evolved and matured (55, 56), balancing the advantages and drawbacks of traditional culture techniques. Since 2009, Hans and colleagues cultivated the first miniature intestinal organoid using adult stem cells derived from the mouse intestine, sparking a surge in organoid research (57). By 2011, Sato and colleagues used organoid technology to establish the first tumor organoids. Based on the culture conditions of mouse colon crypts, they cultured human small intestine and colon organoids and further optimized conditions to establish tumor organoid models from colon adenomas, adenocarcinomas, and Barrett’s esophagus (34). In 2015, Satbir et al. measured cytokine and matrix metalloproteinase (MMP) levels in breast fibroblasts overexpressing inhibitor of growth family member 1(ING1) and co-cultured these cells in three dimensions with Michigan Cancer Foundation - 7(MCF7) cells, finding that matrix ING1 expression could predict survival in ductal-type breast cancer patients, with high levels of ING1 in the matrix cells promoting breast cancer development by increasing MMP expression and downregulating tissue inhibitor of matrix metalloproteinases(TIMPs), potentially leading to reduced patient survival rates (58). In 2016, Nolan E et al. discovered two luminal progenitor subgroups, the receptor of inhibit tumor necrosis factor superfamily member 11(RANK)(+) and RANK(-), in normal tissue of individuals susceptible to breast cancer due to breast cancer susceptibility gene1(BRCA1) mutations, where RANK(+) cells exhibited high proliferative capacity, severe DNA repair abnormalities, and molecular characteristics similar to basal-like breast cancer. They then used denosumab to the inhibit tumor necrosis factor superfamily member 11 (TNFSF11; also known as RANKL) signaling in breast cancer organoids derived from BRCA1 mutation tissue, effectively reducing tumor cell proliferation, identifying a targeted pathway in the primary cell population of BRCA1 mutation carriers, and proposing blocking RANKL as a promising breast cancer prevention strategy (59). In 2021, Eugen et al. focused on the molecular characteristics of tumor cells that persist after treatment, using patient-derived breast cancer organoids to find that persistently treated tumor cells can eliminate drug cytotoxicity by a molecular adaptation similar to embryonic diapause, namely consuming protooncogene Myc or inhibiting the bromodomain protein 4 (Brd4), a Myc transcription coactivator, to eliminate drug cytotoxicity, providing a potential therapeutic strategy for chemotherapy-resistant tumor cells (60). At present, researchers have successfully generated a variety of tumor organoids, including stomach cancer, pancreatic ductal adenocarcinoma, intrahepatic cholangiocarcinoma, gallbladder cancer, esophageal cancer, colon cancer, and breast cancer.

Compared to traditional two-dimensional cultures, organoids represent an innovative technology that can encapsulate the entire physiological process of an organism. They offer a more physiological cell composition and function, more stable genomes, and are better suited for bio-transfection and high-throughput screening. Compared to animal models, organoid models are more streamlined in operation and can also be used to study the mechanisms of disease onset and progression. With the ongoing improvement and development of organoid technology, organoid models have broad application prospects in multiple fields. On one hand, tumor organoid cultures can be used to establish a “living” biological sample bank for drug screening and researching patient gene mutation characteristics. On the other hand, organoid cultures of healthy tissues can be used extensively to study various aspects of tumor initiation and progression, including the effects of pathogens or specific cancer genes. With the inclusion of cellular components of the tumor microenvironment (such as immune cells) into organoid cultures, this technology is now also used in the rapidly evolving field of immuno-oncology (61).

Compared to two-dimensional cell cultures, organoid technology not only allows cells to proliferate at a rate closer to *in vivo* cell proliferation but also better simulates *in vivo* cell morphology and physiology. The sensitivity to drugs and the evaluation of tissue organogenesis is also closer to the cellular conditions under physiological states. Compared to the PDX model, organoid technology eliminates species and individual differences between animal models and humans, reducing ethical risks along with decreased time and economic costs. Compared to the organ tissue slice model, organoid models are better at preserving the potential and complete specificity of tumor stem cells.

Overall, the rapid development and application of organoid technology stems from its unique advantages. First, organoids are structurally and functionally representative. Organoid models can simulate the 3D organ structure, better reflecting the tumor microenvironment *in vivo*, which includes cell–cell interactions and signaling. Second, organoids demonstrate genetic stability. For example, organoid models may be more genetically stable in long-term culture than PDX models. Organoids derived from a patient’s cancer cells are also more personalized and can enable precision therapy. Furthermore, organoids provide a significant advantage in terms of predictability for *in vitro* drug screening, as they may be more accurate in drug screening and therapeutic response prediction due to their ability to better mimic physiological conditions *in vivo*. Finally, organoids facilitate experimental manipulation, reducing the need for complex *in vivo* surgeries and animal experiments, thereby reducing ethical issues and animal welfare concerns. These unique advantages render organoid technology more clinically promising than traditional 2D cell lines, PDX models, and organ slice models.

## 5 Applications of breast cancer organoids

**Figure 6:**
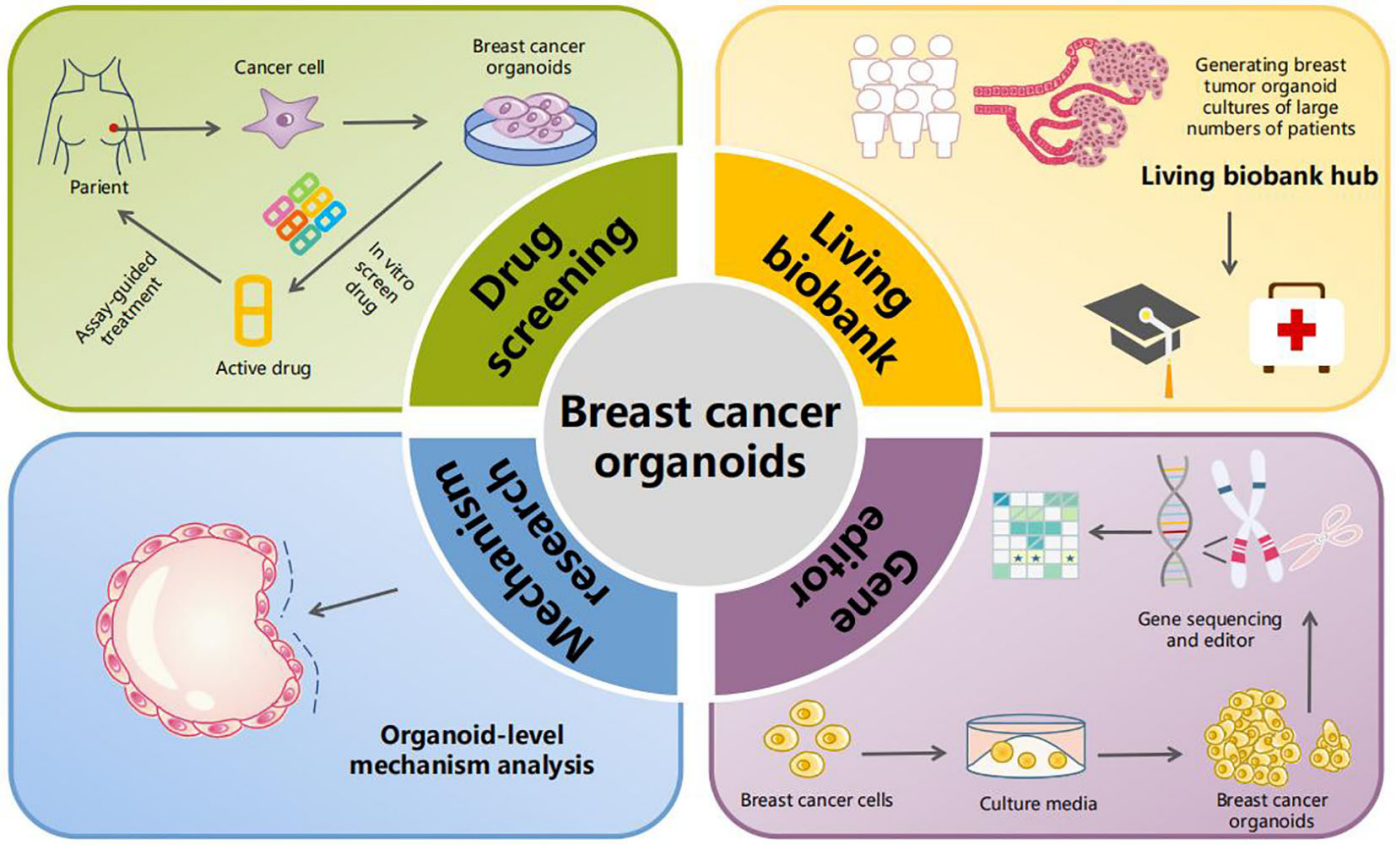
Application of breast cancer organoids. The current applications of breast cancer-like organs involve four main areas. The first is drug screening, where patient-derived breast cancer cells are cultured into organoids that then undergo *in vitro* drug screening, allowing for targeted therapy. The second area is the establishment of breast cancer organoid biobanks. In the third and fourth areas, organoid-level research on tumor mechanisms and gene editing can be conducted.

The keyword clustering mapping demonstrated that the top two keywords were “breast cancer organoids” and “treatment response”. Furthermore, “survival” was a hotspot in the past 2 years among the highly cited keywords. Accordingly, the clinical application of breast cancer organoids is becoming increasingly important. Additionally, “catalytic domain”, “progesterone receptor”, “triple-negative breast cancer”, “specific llama-derived antibodies”, “signaling mediate”, and “mesenchymal stromal cell” (**Figure 3**) demonstrated the mechanistic applications of breast cancer-like organs. **Figure 6** summarizes the current four most important areas of application for breast cancer organoids.

### 5.1 Biobank of “living” breast cancer organoid samples

Researchers have successfully produced many tumor organoids, including but not limited to those from the breast (62), stomach (63), pancreatic duct (64), gallbladder (65), esophagus (34), colon (34), bladder (66), endometrium (67), head and neck (68), kidney (69), liver (70), and lung (71), both primary and metastatic. Breast cancer is composed of multiple distinct subtypes, each with its own genetic, pathological, and clinical characteristics. Hans and colleagues cultured a library of over 100 primary and metastatic breast cancer organoids, confirming that they match the original tumors closely in terms of histopathological subtype, hormone receptor status, and human epidermal growth factor receptor 2(HER2) status. Moreover, to further study tumor development mechanisms and drug screening, they analyzed the breast cancer organoids in-depth using histology, whole-genome deoxyribonucleic acid(DNA) sequencing, ribonucleic acid(RNA) sequencing, and drug tests. They found that the organoids not only closely resemble the histology of the original tumors, displaying phenotypic diversity that can reflect potential mechanisms of cancer histology and identify potential drug targets, but they also maintain genomic stability throughout the culturing process. This allows for easier extraction of genetic data from pure tumor epithelium and detection of somatic mutations (30). The team led by Deng Chuxia generated a breast cancer organoid biobank from tumors with a wide range of treatment statuses, disease stages, and molecular subtypes of breast cancer, achieving a high success rate of up to 75%. The organoids inherited the histological and genetic characteristics of the primary tumors, and the treatments for the primary tumors were well preserved in the corresponding breast cancer organoid models. Predicting patient-specific sensitivity to personalized treatments using drug phenotypes suggests that breast cancer organoid models can serve not only as preclinical models for broader cancer research but also provide personalized treatment recommendations for patients with advanced disease (72).

In summary, establishing a breast cancer organoid biobank has significant advantages for both basic medical research and clinical applications. On the one hand, it can long-term preserve patient samples, facilitating researchers and reducing ethical controversies. On the other hand, once the genetic and epigenetic characteristics of breast cancer organoids are fully confirmed, they can be used for drug screening to develop drugs targeting specific breast cancer features, bringing hope to patients (73).

### 5.2 Drug screening

Preclinical drug development typically involves observing drug efficacy in two-dimensional cell lines to determine clinical candidate drugs, then transitioning to PDX models to study *in vivo* drug effects and safety. However, the cell line models do not truly reflect the patient’s *in vivo* situation. Moreover, PDX cultivation and drug administration cycles are lengthy, limiting their utility in drug screening. The recent advances in organoid technology have effectively bypassed these limitations. Numerous studies have shown that organoids offer higher sensitivity in drug sensitivity monitoring and capture patient heterogeneity, presenting a promising application (74, 75).

Sachs et al. evaluated whether breast cancer organoids can be used for drug screening. They compared the organoids’ response to HER2 pathway inhibitors and poly ADP-ribose polymerase(PARP) inhibitors, finding that HER2-overexpressing organoids were sensitive to anti-HER2 targeted drugs and that BRCAI-mutated breast cancer organoids were sensitive to PARP inhibitors, suggesting that breast cancer organoids can be used to predict the drug response in clinical breast cancer patients (30). Cassidy et al., using breast cancer organoids, screened 18 anticancer drugs. They found that the combination of Palbociclib and Carboplatin or Gemcitabine combined with Erlotinib had a 60% higher cancer suppression rate than other combinations. When applied to patients, the efficacy was superior to current standard treatments (76). Duarte and colleagues used Olaparib-sensitive and -resistant mouse breast cancer organoids for *in vitro* and *in vivo* experiments to test the response to the anticancer drugs Cisplatin and Topotecan. The results showed that organoids lacking p53-binding protein 1(53BP1) derived from Olaparib-resistant mice were less sensitive to both drugs, suggesting that organoid models derived from engineered mouse models can be used to study the responses to various cytotoxic drugs and establish cross-resistance profiles (77). Hence, gene testing of breast cancer organoids and tumor drug screening expand treatment options for patients, promoting individualized precision treatment for tumor patients, and significantly increasing the clinical translation rate. In 2020, Sudhan and others constructed HER2-mutated breast cancer patient-derived organoids to further validate that the combination of neratinib and the Target of Rapamycin Complex 1(TORC1) inhibitor everolimus could significantly inhibit or shrink tumors resistant to neratinib *in vivo*, concluding that the combination of a TORC1 inhibitor with neratinib could be clinically researched in molecular-guided trials of newly diagnosed HER2-mutated breast cancer or acquired mammalian target of rapamycin(mTOR) pathway mutations (78). In 2021, Lu and others identified epigenetic inhibitors GSK-LSD1, CUDC-101, and BML-210 showing antitumor activity in mouse *in situ* breast cancer through high-throughput screening of patient-derived breast cancer organoids. They upregulated major histocompatibility complex class I-mediated antigen presentation in breast tumor cells, and treatment with BML-210 increased the sensitivity of breast cancer organoids to the programmed cell death protein 1(PD-1) (79). In 2022, Yuan Chunhui and others constructed engineered exosomes-HELA-Exos, combining Toll-like receptor 3(TLR3) agonists and immunogenic cell death(ICD) inducers, which can activate dendritic cells *in situ* and specifically induce ICD in breast cancer cells. They verified that in patient-derived breast cancer organoids, HELA-Exos significantly enhanced the generation of conventional type 1 dendritic cells(cDC1) antigen cross-presentation and tumor-reactive CD8+ T cells, indicating that HELA-Exos exhibits potent antitumor activity in breast cancer (80). That same year, Sara and others published a proof-of-concept test case using organoids derived from metastatic breast cancer patients with the Phosphatidylinositol-4,5-Bisphosphate 3-Kinase Catalytic Subunit Alpha(PIK3CA) mutations, assessing treatment with alpelisib (a specific PI3K-α inhibitor) for multiple secondary lesions, providing a new treatment strategy for breast cancer (81). However, the current issue is that drug screening with breast cancer organoids still requires a large amount of clinical research for validation. The representation of tumor microenvironment components (cancer-associated fibroblasts, lymphocytes, macrophages) in epithelial organoids is lacking, preventing a full assessment of the effects of the tumor microenvironment on drug responses. Future validation research will focus more on multi-omics methods, such as metabolomics, proteomics, transcriptomics, etc (81). Professor Zhang Hui’s team used patient-derived organoids (PDOs) and patient-derived xenografts (PDX) to evaluate the rational combination of antitumor activity. Results showed that palbociclib combined with everolimus acted synergistically in ribosomal protein S6 kinase beta 1(S6K1)-amplified breast cancer PDO models, and they proved in PDX models that adding an mTOR inhibitor significantly reduced the levels of phosphorylated retinoblastoma gene(p-Rb) and cyclin D1 in tumors overexpressing S6K1, finding that S6K1 amplification is an important mechanism for inherent resistance of breast cancer to palbociclib (CDK4/6 inhibitor) (82). Another study found high expression of TROJAN in ER+ breast cancer, which by inhibiting cyclin-dependent kinase 2(CDK2) activity, reversed resistance to CDK4/6 inhibitors. *In vitro* experiments showed that a TROJAN LncRNA sensitized breast cancer organoids to the CDK4/6 inhibitor palbociclib, and a series of methods validated the TROJAN-NKRF-CDK2 axis (83).

### 5.3 Research on the mechanism of occurrence and development of breast cancer

The culture of organoids *in vitro* can simulate the growth of tumors in the human body. Through the study of breast cancer organoids, it provides us with new ideas and methods for elucidating tumorigenesis and metastasis. Arruabarrena and colleagues, to further clarify the impact of pioneer transcription factor FOXA1 mutations on the growth of primary mammary cells, introduced WT, Wing2 FOXA1 mutants, and SY242CS mutants into mouse primary mammary organoids using doxycycline-induced lentiviruses. They found that organoids expressing the FOXA1 SY242CS mutation formed larger organoids compared to WT FOXA1, suggesting that SY242CS promotes tumor cell growth (84). Runjie Song and others discovered that circCAPG is highly expressed in clinical samples and cell lines of triple-negative breast cancer (TNBC) and correlates positively with patient survival. To study the function of its translation product CAPG-171aa in TNBC organoids, they confirmed the relationship between CAPG-171aa and serine/threonine kinase 38(STK38) in TNBC through immunoprecipitation and mass spectrometry analyses. Specifically, CAPG-171aa promotes tumor growth by disrupting the binding of to the SMAD-specific E3 ubiquitin-protein ligase 1, thereby preventing MEKK2 ubiquitination and proteasomal degradation (85). During the culture of breast cancer organoids, Cheung et al. found that specialized cancer cells expressing K14 and p63 led to a collective invasion of cancer cells, while K14 knockout tumor organoids were transplanted into mice, and the tumors lost their invasion. Finally, it was proved that the collective invasion behavior is guided by a type of basal-like epithelial cells, which laid the foundation for the use of breast cancer organoids to study tumor metastasis (86). While studying the correlation of protein PIAS1 in human cancer and the mechanism of PIAS1 regulation of breast cancer metastasis, Chanda et al. used organoid culture technology and found that protein inhibitor of activated STAT 1(PIAS1) can inhibit the development of breast cancer cell-derived organoids by regulating the transcriptional regulator Sloan-Kettering Institute novel (SnoN), demonstrating the role of PIAS1 in the mechanism of tumor metastasis (87). Dekkers et al. used CRISPR-Cas9 gene editing technology to target knockout of four breast cancer-related tumor suppressor genes (P53, PTEN, RB1, NF1), transforming healthy breast organoids into breast cancer organoids that can be cultured *in vitro* for a long time. At the same time, 1/6 of the mice implanted with metastatic tumors had p53, PTEN, and RB1 mutations, and 1/2 had p53, PTEN, RB1, and NF1 mutations, and these tumors respond to both chemotherapy and endocrine therapy, improving understanding and awareness of specific molecular subtypes of breast cancer (88).

### 5.4 Breast cancer and gene editing

Organoid culture has been used in two complementary methods. One aims to explore the impact of specific mutations on tumor development by introducing presumed or verified cancer gene mutations into normal organoids or cancer organoids using gene-editing technology. The other involves mutation analysis of patient-derived cancer organoids through whole-genome sequencing (WGS), whole-exome sequencing (WES), or targeted sequencing of cancer gene mutations (61).

The development and refinement of gene editing technologies have made it possible to modify tumor organoids at the genetic level. By using the CRISPR-Cas9 technique, specific DNA modifications can be made to targeted genes in organoids, enabling validation of genes that play a critical role in the development and progression of tumors (89). Davaadelger and colleagues cultivated breast organoids with BRCA1 mutations and normal breast organoids to investigate the effects of hormones and selective progesterone receptor modulators during the menstrual cycle. Their findings revealed that, compared to normal breast organoids, the extracellular matrix of BRCA1 mutated organoids is influenced by estrogen and progesterone. This lays the foundation for future prevention and treatment of breast cancer caused by BRCA1 mutations (90). Deckkers et al. demonstrated the feasibility of using the CRISPR-Cas9 system to genetically engineer tumor suppressor (TS) genes in normal breast epithelial organoids. Through gene-edited organoid cultivation, it was found that breast organoids tend to originate from normal breast epithelial subgroups and can be cultured to produce breast cancer organoids that highly resemble those in the body after gene knockouts (87). Hans and others published an article in “Nature” that employed non-homologous dependent CRISPR-Cas9 technology for rapid and efficient gene knock-in in human-derived organoids, offering an essential platform for endogenous gene knock-ins in human organoids (91). All of this indicates that, in the future, through the CRISPR-Cas9 technique and the continuous development of standardized experimental methods, people can construct breast cancer organoids to identify genes and pathways directly linked to tumor progression, making clinical treatment regimens more precise and effective.

Sequencing of breast cancer organoids can reflect the overall genetics of tumor epithelium, indicating that cancer organoids can represent the heterogeneity of tumors *in vitro* and help find new therapeutic targets. Ding K and others studied the progression and heterogeneity of bone metastases in primary ER+/PR-/HER2- invasive lobular breast cancer using patient-derived organoids (PDOs) from bone metastases progressing to the left pelvis (BoML) and right tibia (BoMR) during letrozole adjuvant therapy. Extensive single-cell RNA sequencing showed that PDOs preserve the subclonal heterogeneity of BoM epithelial cells and show gene expression highly related to BoM, suggesting the use of breast cancer organoids to explore tumor heterogeneity and evolution, as well as precision medicine treatments (92). A study published in “Cancer Research” in 2022 constructed a large-scale metabolic atlas of triple-negative breast cancer (TNBC) by analyzing the polar metabolome and lipidome of 330 TNBC samples and 149 pairs of normal breast tissues. The study divided TNBC into three different subgroups and further confirmed on patient-derived organoid models that targeting the intermediate of the sphingomyelin pathway, sphingosine-1-phosphate, is a promising treatment for luminal androgen receptor breast cancer, advancing the precision treatment of TNBC (93).

Research continues to innovate and develop. The team led by Deng Chuxia found that precision medicine research based solely on genomics and transcriptomics or drug screening based on PDX and PDOs still has significant limitations. Therefore, they combined the advantages of both and proposed the PBPM method—based on pharmacogenomics and ex vivo organoid drug sensitivity models, enabling the genomic and transcriptomic analysis of PDOs to be cross-validated with drug response analysis. Compared to traditional methods, this approach provides higher credibility for treatment recommendations for doctors and patients, with important clinical significance (94).

### 5.5 Precision treatment of breast cancer

Breast cancer is a rapidly developing disease with a poor prognosis, and many questions remain regarding its pathogenesis, early diagnosis, and precise treatment. Organoids are an emerging technology that can simulate the characteristics of breast tumors. Using breast cancer organoid models, it’s possible to study and explore the characteristics of breast cancer, thereby effectively guiding clinical practice and improving patient prognosis. The team led by Shao Zhimin and Jiang Yizhou conducted research on primary tumor tissues and blood samples from 579 patients with luminal breast cancer between 2009 and 2016, drawing a large-scale multi-omics atlas of luminal breast cancer cohorts. Using the similarity network fusion (SNF) algorithm, they precisely classified luminal breast cancer into four different subtypes based on molecular features: classic luminal (SNF1), immune-regulated (SNF2), proliferative (SNF3), and receptor tyrosine kinase (RTK)-driven (SNF4). They demonstrated the effectiveness of “Fudan Quadruple Classification” precision treatment using patient-derived organoids from luminal breast cancer and a retrospective real-world cohort, providing new ideas for precision treatment of breast cancer (95). A 43-year-old TNBC IIA stage patient, after a series of chemotherapy treatments, experienced early metastasis and new bone metastasis after first-line treatment. Drug screening using patient-derived tumor organoids identified eribulin and talazoparib as potential candidate drugs. Subsequent *in vivo* testing with PDOs validated the effectiveness of eribulin, and after obtaining IRB approval, the patient took eribulin, achieving complete remission of liver metastasis. Although there was later progression of bone tumors, and the patient eventually died due to worsening condition, using PDO-informed therapy (eribulin) significantly extended the progression-free survival and time to the next systemic treatment (96). These results demonstrate that functional drug testing with PDOs is feasible and can provide beneficial results in real-time clinical care. However, the robustness, repeatability, and applicability of this testing method for drug (or small molecule) screening with cancer organoids still needs further evaluation before it can be more widely used for personalized medicine approaches (97, 98).

## 6 Limitations and prospects

Breast cancer organoid construction has many advantages in cancer research. It not only allows for the study of the onset and progression of breast cancer *in vitro* and the identification of key tumor genes and pathways through gene editing techniques but also provides a new platform for drug screening, which is expected to solve the problems of personalized medication and precision treatment in clinical practice (99, 100). The organoid culture platform can also simulate immune therapy responses, promoting preclinical testing of immunotherapy (101). Furthermore, more and more innovative ideas based on biomaterials and engineering have been incorporated into traditional organoid culture methods to promote the development of organoid research, enabling better simulation of *in vivo* organ development conditions to produce repeatable and reliable organoids for various applications (102).

However, the application of breast cancer organoid technology also presents challenges. Firstly, constructing organoids is difficult, costly, and may not achieve the desired purity (103). Even though establishing organoid models from surgical or biopsy specimens is feasible, building stable models suitable for biobanking that can grow and replicate long-term is not easy (104).

Secondly, cultivating breast cancer organoids cannot replicate the complex tumor microenvironment structures, such as immune cells, mesenchyme, blood vessels, etc (105–107). Hence, it is challenging to grow them to a larger volume *in vitro*, and it is also impossible to replicate the full functions of the original organ. However, due to the complex composition of the culture matrix gel, which may include unknown growth factors that affect cell viability, there are large differences in the formation efficiency, morphological structure, and function of breast cancer organoid systems, thus limiting the development of breast cancer organoids clinical translational application. Tumor microenvironment includes not only stromal components such as extracellular matrix (ECM), cancer-associated fibroblasts (CAFs), and the vascular system, but also a wide range of immune cells, and together these elements influence tumor progression and treatment response. However, a number of studies in recent years have led to a deeper understanding of the stromal and immune components of the tumor microenvironment. James et al. successfully cultured patient-derived organoids (PDOs) from clinical tumor samples by using an air-liquid interface (ALI) organoid model. This approach was able to maintain the complex organization of the tumor microenvironment (TME), including immune cells within the tumor. Compared with traditional *in vitro* tumor models, this PDO model more realistically reproduces the diversity and physical structure of the TME, providing a more realistic environment for studying immune cell-tumor interactions (108).

Finally, organoids also raise many ethical issues, including controversies related to informed consent and ownership of human embryo storage and the development of biobanks. Currently, efforts are being made to utilize automation and optimized cultivation schemes to improve the success rate and efficiency of breast cancer organoid construction. It is believed that as organoid technology advances, these limitations will be progressively overcome, playing an even more prominent role in clinical applications, new drug development, and basic research, offering new therapeutic hopes for patients with limited treatment options (109).

Organoid culture will likely be facilitated by the application of bio-3D printing (110). Bio-3D printing would yield many advantages for organoid construction. First, it achieves fully automated, high-throughput production, which can construct numerous organoid models quickly and efficiently, improving productivity. Second, bio-3D printing is highly stable and reproducible, which ensures that each printed organoid model has the same characteristics and performance and enhances the reliability of experimental results. Additionally, bio-3D printing can construct complex and controllable organoid structures, accurately controlling cell arrangement and tissue structure, bringing organoids closer to real biological tissues, and improving the simulation of the models. In conclusion, 3D printing technology offers more possibilities for organoid technology development, promising to improve current organ models and improve biomedical research and applications (111–113).

In summary, organoids cultured from tumor tissues sourced from breast cancer patients can be applied to study tumor development, simulate breast cancer diseases (114), and have played a pivotal role in establishing breast cancer organoid biobanks, personalized medicine for antitumor drug screening, and gene therapy combined with genome editing technology. At present, the application of breast cancer organoid technology is in its infancy. Extensive research will enable 3D organoid systems to fill the gaps present in current 2D cell line models and animal models, making them a research hotspot in the field of breast cancer basic research and clinical applications in the future.

## Data Availability

The original contributions presented in the study are included in the article/supplementary material. Further inquiries can be directed to the corresponding authors.

## Glossary

HER2: Human Epidermal Growth Factor Receptor 2
WOSCC: Web of Science Core Collection
3D: Three-dimensional
2D: Two-dimensional
EGF: Epidermal growth factor
NGS: Next-Generation Sequencing
CNV: Copy number variants
SNV: Single nucleotide variants
MAPK: Mitogen-activated protein kinase
CRC: Colorectal cancer
PDX: The patient-derived xenograf
PDOs: Patient-derived organoids
ECM: Extracellular matrix
MMP: Matrix metalloproteinase
ING1: Inhibitor of growth family member 1
TIMPs: Tissue inhibitor of matrix metalloproteinases
BRCA1: Breast cancer susceptibility gene1
RANK: The receptor of inhibit tumor necrosis factor superfamily member 11(TNFSF11A
RANKL: The inhibit tumor necrosis factor superfamily member 11 (TNFSF11
Brd4: The bromodomain protein 4
PARP: Poly ADP-ribose polymerase
53BP1: P53-binding protein 1
TORC1: Target of Rapamycin Complex 1
mTOR: Mammalian target of rapamycin
PD-1: Programmed cell death protein 1
TLR3: **Toll-like receptor 3;** ICD, Immunogenic cell death
cDC1: Conventional type 1 dendritic cells
PIK3CA: Phosphatidylinositol-4,5-Bisphosphate 3-Kinase Catalytic Subunit Alpha
S6K1: Ribosomal protein S6 kinase beta 1
p-Rb: Phosphorylated retinoblastoma gene
CDK2: Cyclin-dependent kinase 2
TNBC: Triple-negative breast cancer
STK38: Serine/threonine kinase 38
PIAS1: Protein inhibitor of activated STAT 1
WGS: Whole-genome sequencing
WES: Whole-exome sequencing
ER: Estrogen Receptor
PR: Progesterone receptor
SNF: Similarity network fusion
CAFs: Cancer-associated fibroblasts
ALI: Air-liquid interface.
